# Successful treatment of cutaneous siderosis using a 1064‐nm Nd:YAG picosecond laser following intravenous iron extravasation: A case report

**DOI:** 10.1111/jocd.16416

**Published:** 2024-05-30

**Authors:** Whiin Lee, Yun Su Eun, Jae Yun Kim, Sul Hee Lee, Youngsook Kim, Jung Eun Kim

**Affiliations:** ^1^ Department of Dermatology Soonchunhyang University Cheonan Hospital Cheonan South Korea; ^2^ Department of Dermatology Soonchunhyang University Bucheon Hospital Bucheon South Korea; ^3^ Division of Endocrinology and Metabolism, Department of Internal Medicine Yonsei University College of Medicine Seoul South Korea

To the Editor,

Postpartum iron deficiency anemia is a significant maternal comorbidity and has been associated with maternal impaired cognition, depression, tiredness, and infections, particularly in the urinary tract. Intravenous iron replacement has advantages that bypass absorption challenges and rapidly replenish iron stores and, thus, is currently considered a relatively effective and safe treatment for postpartum iron‐deficiency anemia. However, it carries potential adverse effects, such as reactions related to infusion, hypophosphatemia, and skin staining.^1^ One specific complication observed is the development of a gray to brown skin pigmentation, known as cutaneous siderosis, resulting from the extravasation of iron solutions. There is a significant demand for effective treatments among patients suffering from this complication, as the hyperpigmentation can be permanent. However, the currently reported treatment options are notably limited.

We report the case of a 35‐year‐old female with Fitzpatrick skin type III who experienced this complication. She had been treated for postpartum anemia with intravenous ferric carboxymaltose (Ferinject; Vifor, St. Gallen, Switzerland) once daily for two consecutive days. During the second session of treatment, she experienced paravenous infusion of iron solution. This resulted in a grayish to brownish discoloration on the forearm, which persisted for 1 month. Dermatological evaluation revealed a 10 × 15 cm sized extensive hyperpigmentation with irregular border extending from the elbow crease to the forearm (Figure 1A). A skin biopsy from the hyperpigmented skin lesion revealed aggregates of brown granules indicative of iron deposits, primarily surrounding vascular structures in both superficial and deep dermal layers (Figure 2A). When subjected to iron staining, these granules appeared blue, confirming the presence of iron deposits (Figure 2B). Finally, a diagnosis of cutaneous siderosis was done.

**FIGURE 1:**
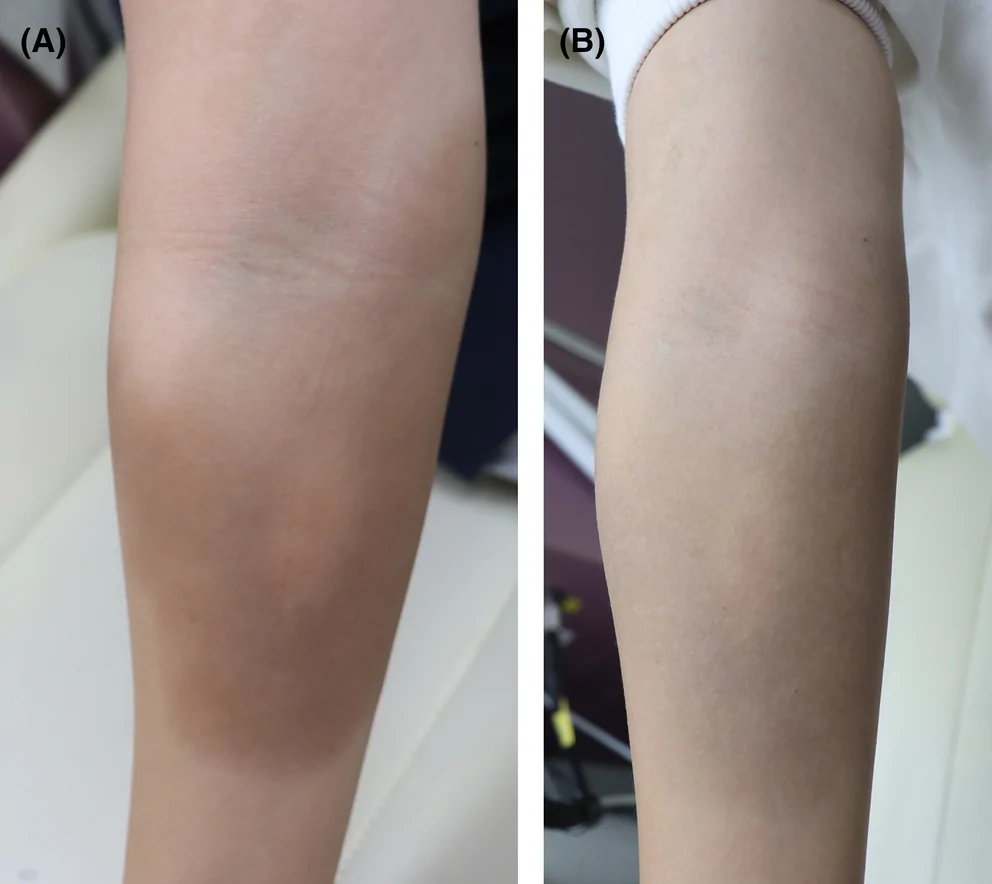
Treatment of cutaneous siderosis with a 1064‐nm Nd: YAG picosecond laser. (A) A 10 × 15 cm sized hyperpigmented skin lesion. (B) After 10 treatment session with a 1064‐nm Nd: YAG picosecond laser.

**FIGURE 2:**
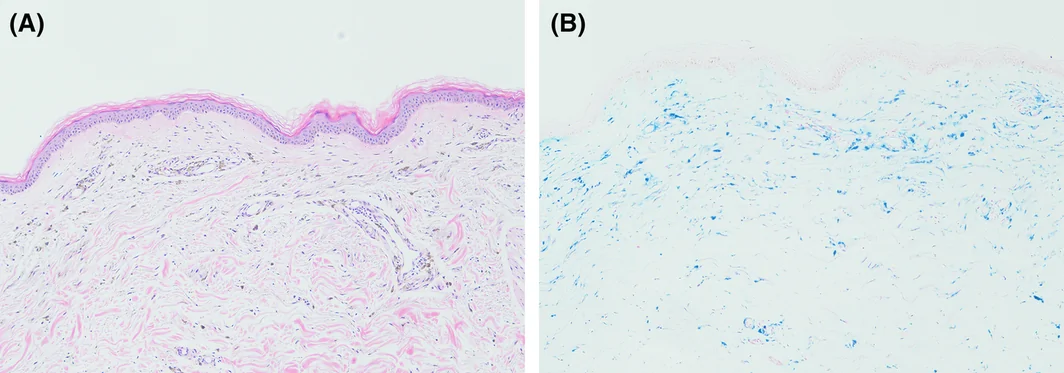
(A) Clusted brown granules consistent with iron pigmentation were seen mostly around the vessels in superficial and deep dermis. (B) Iron staining showed these granules as blue, which is a sign of iron deposit.

Due to cosmetic concerns, we initiated treatment about 3 weeks later with a 1064‐nm Nd:YAG picosecond laser (Picoway; Candela Corp., Marlborough, MA, USA), administered at monthly intervals. The treatment settings included a fluence of 1.2 J/cm^2^, a spot size of 4 mm, and a repetition rate of 10 Hz. Following treatment, slight erythema and immediate petechiae were noted on the treated skin, which resolved within a week. Posttreatment care involved cooling the treated areas with icepacks and applying topical desonide cream. After undergoing 10 treatment sessions, there was a significant improvement in the hyperpigmentation (Figure 1B), with no scarring or dyschromia observed. The patient expressed satisfaction with the cosmetic results.

Extravasation of intravenous iron at the site of infusion leads to the iron accumulation in the dermis, resulting in persistent hyperpigmentation that ranges from grayish to brown, known as cutaneous siderosis. Ferinject, containing ferric carboxymaltose, is a contemporary iron (III)‐hydroxide carbohydrate complex intended for intravenous application. While rare, extravasation at the injection site has been reported, with the highest incidence observed at 2.2% in a study involving 139 patients with chronic kidney disease not requiring dialysis.^2^ Previous reports have described the treatment of cutaneous siderosis using various lasers, including the 755‐nm Q‐switched (QS) alexandrite laser, the 532‐nm QS Nd:YAG laser, and the 964‐nm QS ruby laser.^3^, ^4^ The emergence of picosecond laser technology has marked a new era in treating diverse dermal pigment disorders, including acquired bilateral nevus of Ota‐like macules and post‐inflammatory hyperpigmentation.^5^, ^6^ However, to the best of our knowledge, there are no reports of a laser protocol similar to ours, which treats cutaneous siderosis with multiple sessions using only the 1064‐nm Nd:YAG picosecond laser at a low fluence.

Cutaneous siderosis, although uncommon, results in distinctive and lasting hyperpigmentation. We propose that the 1064‐nm Nd:YAG picosecond laser offers a novel, efficient, and secure modality for treating this condition. Further research is required to understand the precise mechanism of action of the 1064‐nm Nd:YAG picosecond laser in treating cutaneous siderosis.

## AUTHOR CONTRIBUTIONS

*Conceptualization*: Jung Eun Kim. *Formal analysis*: Whiin Lee. *Investigation*: Yun Su Eun, Jae Yun Kim, Sul Hee Lee, Youngsook Kim. *Writing‐original draft*: Whiin Lee. *Writing‐review and editing*: Jung Eun Kim.

## FUNDING INFORMATION

None.

## CONFLICT OF INTEREST STATEMENT

None declared.

## ETHICS STATEMENT

Patient provided informed consent for the disclosure of details and images of case.

## Data Availability

The data that support the findings of this study are available from the corresponding author upon reasonable request.
